# Correction: Kidney damage on fertility and pregnancy: A Mendelian randomization

**DOI:** 10.1371/journal.pone.0318830

**Published:** 2025-01-30

**Authors:** Jin Ren, Qiuyan Huang, Xiaowei Nie, Xingli Tong, Qi Yao, Ge Zhou

The third author’s name is spelled incorrectly. The correct name is: Xiaowei Nie. The correct citation is: Ren J, Huang Q, Nie X, Tong X, Yao Q, Zhou G (2023) Kidney damage on fertility and pregnancy: A Mendelian randomization. PLoS ONE 18(7): e0288788. https://doi.org/10.1371/journal.pone.0288788.
